# Double renal cell carcinoma with histological type of clear cell carcinoma and papillary carcinoma in the same kidney concurrently treated with robot‐assisted partial nephrectomy

**DOI:** 10.1002/iju5.12395

**Published:** 2021-12-02

**Authors:** Teruki Shimizu, Fumiya Hongo, Hikaru Takahashi, Atsuko Fujihara, Osamu Ukimura

**Affiliations:** ^1^ Department of Urology Kyoto Prefectural University of Medicine Kyoto Japan

**Keywords:** clear cell renal carcinoma, ipsilateral, papillary type1 renal cell carcinoma, robot‐assisted partial nephrectomy

## Abstract

**Introduction:**

The presence of two different histologic types of renal cell carcinoma in the same kidney is rare in clinical practice. This report describes a patient with ipsilateral renal cell carcinomas, consisting of a clear cell renal cell carcinoma and a papillary type 1 renal cell carcinoma, who was successfully treated by robot‐assisted partial nephrectomy.

**Case presentation:**

A 70‐year man was referred to our hospital for the treatment of two right mid‐pole renal tumors, measuring 51 mm and 31 mm in diameter. The two tumors, which differed in contrast enhancement on computed tomography, were removed simultaneously by robot‐assisted partial nephrectomy. Histopathological and immunohistochemical findings confirmed that one tumor was a clear cell renal cell carcinoma, pT1a, and the other was a papillary type1 renal cell carcinoma, pT1b.

**Conclusion:**

This report describes a rare patient presenting with two ipsilateral renal cell carcinomas differing in histology. Robot‐assisted partial nephrectomy was the safe and effective nephron‐sparing surgery, even in patients with complex double renal tumors.

**Abbreviations & Acronyms**
ccRCC: clear cell RCC
CT: computed tomography
pRCC: papillary RCC
RAPN: robot‐assisted partial nephrectomy
RCC: renal cell carcinoma
WIT: warm ischemia time

**Keynote message**

Synchronous occurring ipsilateral RCCs differing in histology are rare. RAPN has proven safe and useful even in patients with complex double renal tumors. In patients with synchronous occurring ipsilateral double RCCs, RAPN would be one of the feasible treatment options in minimally invasiveness as well as renal function preservation.

## Introduction

RCC is the most common malignancy of the kidney. However, synchronous ipsilateral RCCs differing in histology are extremely rare, both clinically and pathologically. RAPN is a minimally invasive type of nephron‐sparing surgery for patients with small renal masses. This report describes a patient with unilateral synchronous RCCs, consisting of papillary type1 classified as cT1bN0M0 and clear cell type classified as cT1aN0M0, which were successfully removed by RAPN at once with negative surgical margins.

## Case presentation

**Fig. 1:**
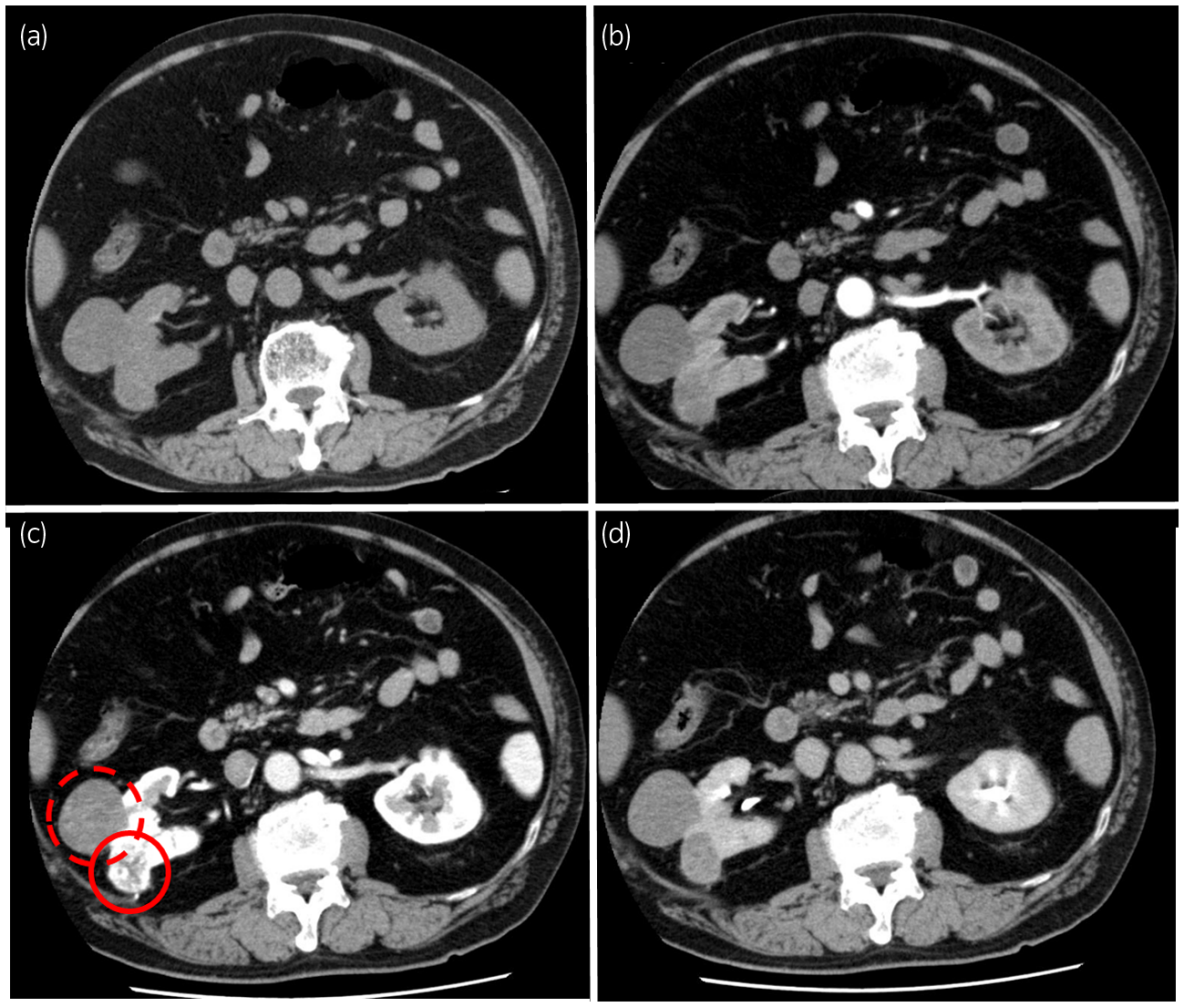
CT enhancement patterns of the two ipsilateral renal tumors in this patient. (a) Plain CT, (b) early arterial phase, (c) parenchymal phase, and (d) delayed phase. The cT1a renal tumor (red circle) showed hypervascularity on the parenchymal phase, whereas the cT1b renal tumor (dotted red circle) showed low vascularity indicated by scarce contrast enhancement.

**Fig. 2:**
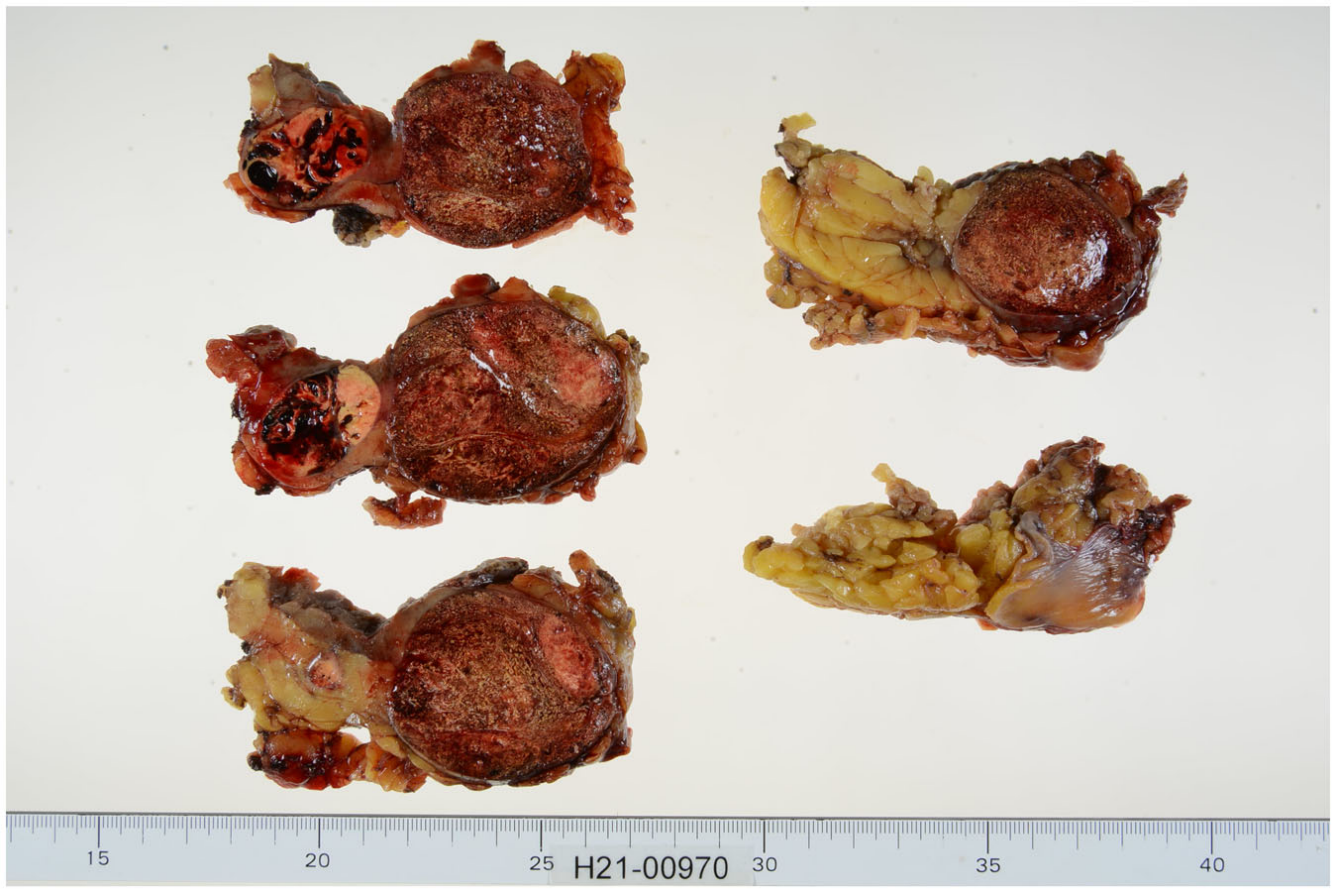
Macroscopic characterization of the two renal tumors. The larger tumor was a vaguely circumscribed solid mass, measuring 51*45 mm in diameter, whereas the smaller tumor was a well‐circumscribed solid mass, measuring 31*21 mm in diameter. The surface cut was yellow with central hemorrhage.

**Fig. 3:**
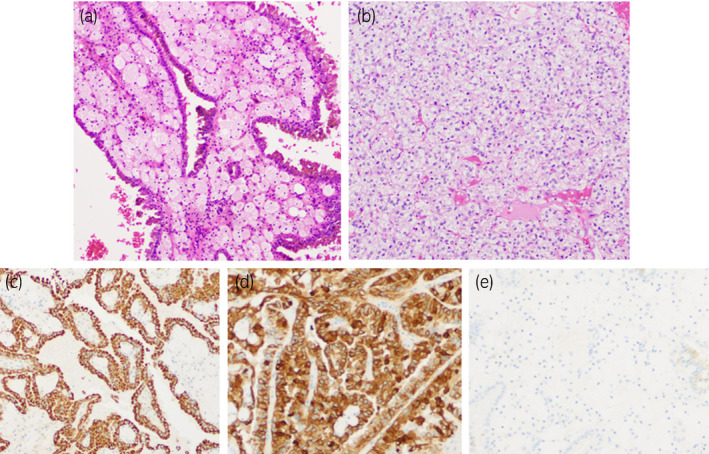
Microscopic characterization of the two renal tumors. (a) The larger tumor showed papillary architecture in general, with inner hemorrhage and hyalinization, diagnosed as papillary type1 RCC (H&E staining). (b) The smaller tumor showed clear cytoplasm and stroma, and was diagnosed as a clear cell RCC (H&E staining). (c‐e) Immunohistochemical staining of sections of the larger tumor with antibodies to (c) AMACA, (d) CK‐7, and (e) CA9. Sections were positive for AMACA and CK‐7, but negative for CA9, results consistent with papillary type 1 RCC.

A 70‐year‐old man was referred to our hospital for the treatment of two renal masses on the right kidney that had been incidentally detected on CT scanning performed to evaluate lower abdominal discomfort. The contrast‐enhanced CT scanning revealed two renal masses on his right kidney. One lesion, 50 mm in diameter and classified as cT1bN0M0, was detected at the mid‐kidney and the second lesion, 30 mm in diameter and classified as cT1aN0M0, was present at the mid‐kidney near the first lesion. The two tumors differed in contrast staining patterns, with the T1b lesion having little contrast enhancement and the T1a lesion showing early contrast enhancement followed by late phase washout of the contrast agent (Fig. 1). Because these two tumors were located near each other at the mid‐pole of the right kidney, the intention was to remove both at the same time. RAPN was performed via the transperitoneal approach. The two renal tumors were removed simultaneously (combined weight, 161 g). Because the two tumors were large to remove at once, extensive dissection was required to mobilize the kidney, the total console time was relatively long (303 min), the WIT was 15 min, inner suturing, the early unclamping method followed by renal parenchymal suturing and the estimated blood loss was 400 mL. Macroscopic examination of the resected specimen showed a vaguely circumscribed solid mass, 51 mm in diameter, at the mid‐pole of the kidney, and a smaller, well‐circumscribed, solid mass, 31 mm in diameter, near the first tumor. The surface cut of the tumor was yellow with central hemorrhage (Fig. 2). Microscopically, hematoxylin and eosin (H&E) stained sections of the larger tumor showed a papillary architecture with inner hemorrhage and hyalinization; this tumor was diagnosed as a type1 papillary RCC, pT1b, with negative surgical margins. H&E staining of the smaller tumor showed an acinar architecture with clear cytoplasm and stroma; this tumor was diagnosed as a clear cell RCC, pT1a, with negative surgical margins. Because these two tumors did not have a continuous border, they were diagnosed as a double cancer of the same kidney. Immunohistochemical staining of the larger tumor showed that the tumor cells were positive for AMACA and CK‐7, but negative for CA9 (Fig. 3), further indicating that the larger tumor was papillary type1 RCC. Follow‐up CT 3 months after surgery showed no clinical signs of local recurrence or metastasis. His preoperative and postoperative estimated glomerular filtration rates (eGFR) were 79.6 mL/min and 70.2 mL/min, respectively.

## Discussion

Types of RCC encountered in clinical settings include ccRCC, which is present in 70% of patients, followed by pRCC, which is present in about 10%.^1^ Papillary RCCs are classified into types1 and 2 based on their morphologic appearance.^2^

Although very rare, ipsilateral renal lesions differing in RCC histology have been reported.^3^, ^4^ For example, a study of 16 patients with ipsilateral synchronous RCC found that the most common histological combination was ccRCC and chromophobe RCC, observed in three patients, pairing of ccRCC and pRCC was observed in two patients. Of these 16 patients, nine underwent radical nephrectomy and six underwent partial nephrectomy. Evaluation of oncological outcomes showed that three patients developed distant metastases after surgery, one after partial nephrectomy, and two after radical nephrectomy.^4^

We encountered a rare patient with two different RCCs coexisting in the same kidney. Although ipsilateral multifocal RCCs are under controversial regarding indication of radical nephrectomy or partial nephrectomy, patients receiving radical nephrectomy inevitably suffer from post‐surgical renal function decline. Fortunately, the two tumors in our patient were located near each other in the mid‐kidney, suggesting that partial nephrectomy would be sufficient to simultaneously remove both tumors. Importantly, the comparative advantages and disadvantages of radical nephrectomy and partial nephrectomy, including oncological outcomes and post‐surgical renal function, should be determined in each patient based on factors such as tumor (ⅰ) size (ⅱ) location (ⅲ) presumable pathology. In more challenging cases of ipsilateral synchronous renal tumors, simply speaking, if sufficient tumor negative margin is expected to be difficult, it may be a more feasible strategy to remove the larger tumor by partial nephrectomy and to treat the smaller tumor with focal ablation therapies, including cryoablation or radiofrequency ablation.

Both macroscopic and microscopic findings showed that the T1a lesion in our patient was a typical ccRCC, whereas the T1b lesion was not. Histopathological and immunohistochemical staining revealed that the T1b lesion was a pRCC. Pathological characterization has shown that pRCC is less invasive than ccRCC, and patients with pRCC have been reported to have better oncological outcomes than patients with ccRCC.^5^ Recent guidelines indicate that T1a‐b RCC is associated with a low risk of post‐surgical tumor recurrence and recommend that CT surveillance be discontinued more than 5 years after surgery.^6^ However, recurrences in over 50% of patients with pathologic T1a have been found to occur more than 5 years after surgery.^5^ Moreover, rates of contralateral metachronous recurrence have been reported to be fivefold higher in patients with multiple ipsilateral renal tumors than in patients with solitary renal masses.^7^ These findings indicate that our patient should be carefully followed‐up for long periods of time.

The two tumors in our patient showed different enhancement patterns on CT, suggesting that differences in enhancement patterns may allow the detection of tumors of different histology. As our case maintained renal function even after radical renal surgery, availability to use contrast agent would be an important factor also in follow‐up. This view seems important because different histological types of RCC differ in aggressiveness and prognosis.

## Conclusion

Multiple ipsilateral renal masses that differ histologically are a very rare entity among patients undergoing partial nephrectomy. This report describes a patient with synchronous ccRCC and pRCC in the same kidney who was successfully treated by a single RAPN, with negative surgical margins as well as well‐preservation of renal function.

## Conflict of interest

The authors declare no conflict of interest.

## Approval of the research protocol by an institutional reviewer board

Not applicable.

## Informed consent

Informed consent for publication was obtained from the patient.

## Registry and the registration no. of the study/trial

Not applicable.
